# Prognostic value of longitudinal antinuclear antibody dynamics in rheumatoid arthritis: a retrospective cohort study

**DOI:** 10.3389/fimmu.2026.1743637

**Published:** 2026-04-21

**Authors:** Xinyu Li, Yuan Li, Yan Ma, Rui Bu, Xuesong Liu, Qianqian Li, Liangjing Lu

**Affiliations:** 1 Department of Rheumatology, Renji Hospital, Shanghai Jiao Tong University School of Medicine, Shanghai, China; 2 Department of Rheumatology, The Affiliated Suzhou Hospital of Nanjing Medical University, Suzhou Municipal Hospital, Suzhou, Jiangsu, China; 3 Department of Ultrasound, Renji Hospital, School of Medicine, Shanghai Jiao Tong University, Shanghai, China

**Keywords:** ANA dynamics, antinuclear antibodies, DAS28-CRP, remission, rheumatoid arthritis, targeted synthetic DMARDs, TNF inhibitor

## Abstract

**Background:**

The prognostic value of longitudinal antinuclear antibody (ANA) dynamics in rheumatoid arthritis (RA) remains unclear.

**Objectives:**

To examine whether ANA development (titer rise/seroconversion) is associated with 12-month clinical remission and to develop a prediction model for DAS28-CRP remission.

**Methods:**

We retrospectively enrolled 688 adults with RA (2010 criteria) from 2016–2023. ANA was assessed at baseline and 6 months (± 2 months); development was defined as a ≥1-dilution increase or seroconversion (<1:80 to ≥1:80). The primary endpoint was DAS28-CRP remission at 12 months (<2.6); other endpoints were secondary/exploratory. We used propensity score matching where appropriate. Multivariable logistic regression identified predictors, with performance assessed by AUC and calibration. Subgroup analyses distinguished seroconversion from titer elevation, and were stratified by baseline RF/anti-CCP status. Sensitivity analyses used a higher positivity threshold (≥1:160).

**Results:**

Baseline ANA positivity was 63.7%, typically low titers (1:80, 1:160). Among 467 patients with serial ANA data, 94 (20.1%) exhibited ANA development, which was associated with significantly higher post-treatment disease activity and lower remission rates across multiple criteria (e.g., DAS28-CRP 43.8% vs 65.2%, p=0.004). In multivariable analysis, ANA development independently predicted non-remission (OR 0.472, p=0.010) together with baseline DAS28-CRP (OR 0.745, p=0.017). The prediction model achieved moderate discriminative ability, with an area under the curve (AUC) of 0.715 in the training cohort and 0.705 in the testing cohort, alongside acceptable calibration. Stratified analysis revealed that the negative prognostic value of ANA development was most pronounced in RF-negative patients (adjusted OR = 0.29, p=0.048). Intriguingly, baseline homogeneous ANA pattern was associated with higher remission rates (63.8% vs. 41.5% in pure speckled pattern, p<0.001). Sensitivity analysis using a ≥1:160 cutoff yielded distinct findings: high ANA titer was associated with a higher likelihood of remission (OR = 1.624, p=0.016).

**Conclusions:**

Rising ANA titers/seroconversion during therapy are associated with reduced probability of DAS28-CRP remission at 12 months. The prognostic impact is modulated by baseline RF status, ANA fluorescence patterns, and the titer cutoff used. Conversely, a high baseline ANA titer (≥1:160) itself may identify a subgroup with distinct characteristics. Incorporating ANA dynamics into routine monitoring may improve risk stratification and clinical decision-making in RA.

## Introduction

Rheumatoid arthritis (RA) is a chronic systemic autoimmune disease characterized by persistent synovial inflammation, which leads to joint destruction and disability (1). It affects approximately 0.5 - 1% of the global population, with substantial geographic and ethnic variation, and is more common in women (1, 2). RA is clinically heterogeneous, with considerable variation in symptoms, disease progression, and treatment response (3–5). This heterogeneity has prompted the search for biomarkers that can help predict disease outcomes and guide individualized treatment. Currently, commonly used serological markers include rheumatoid factor (RF) and anti-cyclic citrullinated peptide (anti-CCP) antibodies, both of which are included in the 2010 ACR/EULAR classification criteria (6). Other autoantibodies, such as anti-peptidylarginine deiminase 4 (anti-PAD4) antibodies (7, 8) and anti-mutated citrullinated vimentin (anti-MCV) antibodies (9), have been studied but lack consistent clinical utility. Notably, reliable biomarkers predictive of treatment response or adverse reactions remain an unmet need in RA.

Antinuclear antibodies (ANA) are a group of autoantibodies directed against nuclear and cytoplasmic components (10). ANA positivity is a hallmark of systemic autoimmune diseases such as systemic lupus erythematosus (SLE) (11), systemic sclerosis, and mixed connective tissue disease (12), but can also be seen in organ-specific autoimmune diseases (13) and even in healthy individuals (14). ANA levels often rise before disease onset and decline with disease control, making them useful for early diagnosis and disease monitoring in connective tissue diseases such as SLE (12). However, such associations are less clearly supported in RA, where the role of ANA dynamics in disease monitoring remains a subject of ongoing investigation.

In RA, ANA positivity is reported in 30–60% of patients, usually at low titers, and is often associated with homogeneous or speckled staining patterns (15). While ANA testing may enhance diagnostic sensitivity when combined with RF and anti-CCP, most studies have not shown a strong link between ANA and joint disease activity (16). ANA-positive RA patients with ANA positivity have been reported to show more frequent joint involvement (17) and an increased risk of bone erosion (18). Nevertheless, findings regarding the clinical significance of ANA in RA have been inconsistent. For instance, tumor necrosis factor inhibitors (TNFi) (19) and other biologic DMARDs (bDMARDs) (20) have been reported to induce elevations in ANA and other autoantibodies, which are sometimes associated with an unfavorable prognosis. Critically, however, whether such treatment-induced ANA development holds predictive value for clinical outcomes remains poorly defined. It is crucial to distinguish whether ANA development represents treatment-induced autoantibody production (e.g., associated with specific biologics) or mirrors underlying disease-driven immunologic activation, as these two mechanisms may carry distinct prognostic implications.

This study aimed to (1) characterize the clinical features of ANA-positive RA patients and those with ANA development during treatment; (2) evaluate the association between ANA positivity—particularly titer elevation—and disease prognosis; and (3) develop a predictive model for long-term treatment response. By examining longitudinal changes in ANA titers, we seek to clarify their prognostic value and potential utility in guiding personalized treatment strategies.

## Methods

### Study population

This retrospective cohort study included patients diagnosed with RA according to the 2010 American College of Rheumatology/European League Against Rheumatism (ACR/EULAR) classification criteria. Eligible patients were consecutively enrolled from the Rheumatology Department of Renji Hospital, Shanghai Jiao Tong University School of Medicine, between January 1, 2016, and December 31, 2023. Exclusion criteria were as follows: (1) age <18 or >90 years; (2) coexisting autoimmune diseases; (3) history of benign or malignant neoplasms; (4) active infections (e.g., tuberculosis or hepatitis B); (5) pregnancy or lactation; and (6) recipients of organ transplantation. Patients were followed for at least 12 months. ANA testing was performed at both baseline and follow-up, and individuals with incomplete clinical data were excluded.

### Data collection

At the initial visit, demographic and clinical data were collected using a standardized questionnaire, including age, BMI, smoking and alcohol history, disease duration, and prior treatment history. Laboratory parameters included complete blood count, erythrocyte sedimentation rate (ESR), rheumatoid factor (RF), anti-CCP antibodies, immunoglobulin levels, complement components (C3/C4), liver and renal function tests, and a comprehensive autoantibody panel, including ANA. A senior rheumatologist conducted a comprehensive clinical evaluation, including joint counts (TJC28, SJC28), assessment of extra-articular involvement (e.g., pulmonary, cardiac, ophthalmologic, hematologic, hepatic, renal), and administration of standardized instruments: visual analog scale (VAS), Health Assessment Questionnaire-Disability Index (HAQ-DI), and EQ-5D. Disease activity was evaluated using The Disease Activity Score 28 (DAS28), and the simplified and clinical disease activity indices (SDAI, CDAI). Treatment data, including type and dosage of disease-modifying anti-rheumatic drugs (DMARDs) and corticosteroids, were collected at baseline and follow-up. Treatment response was assessed at the 12-month follow-up. This study was reviewed and approved by the Ethics Committee of Renji Hospital, Shanghai Jiao Tong University School of Medicine (approval No. RA-2022-538). All participants gave written informed consent.

Multiple metrics were used to define treatment response (21): (1) Remission was defined as DAS28-ESR <2.6, DAS28-CRP <2.6, SDAI <3.3, or CDAI <2.8. (2) EULAR response criteria were applied only to patients with moderate-to-high disease activity at baseline to avoid ceiling effects. A good response was defined as an improvement in DAS28 >1.2 with an endpoint score ≤3.2. A non-response was defined as DAS28 ≤0.6 or DAS28 ≤1.2 with a final DAS28 >5.1. Intermediate responses were classified accordingly. (3) Boolean 1.0 remission: TJC ≤1, SJC ≤1, CRP ≤1 mg/dL, PtGA ≤1; Boolean 2.0: CRP ≤2 mg/dL, PtGA ≤2. (4) ACR20/50/70 responses required ≥20%, ≥50%, or ≥70% improvement in TJC and SJC, and ≥20%/50%/70% improvement in 3 out of 5 domains (VAS, PtGA, PhGA, HAQ-DI, acute phase reactants). (5) HAQ improvement was defined as a reduction in HAQ-DI of ≥0.25 from baseline. DAS28-CRP remission (DAS28-CRP < 2.6 at 12 months) was pre-specified as the sole modeling endpoint based on data completeness, measurement robustness, and clinical interpretability. ANA measurement and definition of ANA development. ANA was measured at a single centralized, certified laboratory at Renji Hospital at baseline and 6 months (± 2 months). Throughout the study period (2016–2023), testing was consistently performed using the same indirect immunofluorescence (IIF) assay on HEp-2 cells (Euroimmun, Lübeck, Germany). To ensure longitudinal methodological consistency and minimize inter-assay variability, the laboratory adhered to strict standard operating procedures (SOPs), including routine internal quality control and external quality assessment. Fluorescence patterns and titers were interpreted by experienced laboratory personnel blinded to the patients’ clinical data. ANA development was *a priori* defined as (i) a rise of ≥1 serial dilution from baseline (e.g., 1:80→≥1:160) or (ii) seroconversion from negative (<1:80) to positive (≥1:80). Clinical endpoints were evaluated at 12 months, ensuring a fixed temporal sequence (exposure at ~6 months; outcome at 12 months). Sensitivity analyses used a stricter definition (≥2 dilutions or threshold ≥1:160).

### Statistical analysis

To minimize potential confounding due to baseline differences (e.g., age, sex, BMI, and age at onset), propensity score matching (PSM) was performed using logistic regression and 1:1 nearest neighbor matching with a caliper of 0.02. Continuous variables were tested for normality. Normally distributed variables were expressed as mean ± SD, while skewed distributions were reported as median (IQR). Categorical variables were summarized as frequencies (percentages). Between-group comparisons were performed using the t-test or Mann–Whitney U test for continuous variables and Pearson’s χ², Yates correction, or Fisher’s exact test for categorical variables. Ordinal variables were compared using the Mann–Whitney U test. Spearman correlation was used for correlation analysis.

Univariate and multivariate logistic regression analyses were used to identify prognostic factors. A stepwise regression model was constructed, and model performance was evaluated by receiver operating characteristic (ROC) curve analysis (AUC), Hosmer–Lemeshow goodness-of-fit test, and calibration plots. For ANA pattern analysis, we created mutually exclusive groups to account for patients with multiple patterns. Stratified analyses by baseline RF and anti-CCP status were performed using multivariate logistic regression within each stratum. A sensitivity analysis redefining clinically significant ANA positivity with a cutoff of ≥1:160 was conducted to test the robustness of the primary findings. All statistical analyses were conducted using IBM SPSS version 26.0, with a significance level of α = 0.05 (two-sided). Figures were generated using GraphPad Prism 9.5.

## Results

### Baseline ANA positivity and clinical features

A total of 688 patients with rheumatoid arthritis (RA) were included in the baseline analysis. The overall ANA positivity rate was 63.7%. Among ANA-positive individuals, the most common titers were 1:80 (n = 218, 49.8%) and 1:160 (n = 71, 16.7%). Moderate titers of 1:320 and 1:640 were observed in 17.8% and 9.4% of patients, respectively, while high titers (≥1:1280) were identified in 6.4% (n = 28). The predominant ANA staining patterns were homogeneous (31.4%) and speckled (26.9%), with a mixed homogeneous/speckled pattern observed in 4.9% of patients (**Supplementary Figure 1**). Among specific autoantibodies, anti-Ro60 and anti-Ro52 were the most frequently detected, with positivity rates of 16.0% and 15.4%, respectively (**Supplementary Figure 2**).

Patients were classified into ANA-positive (titer ≥1:80, n = 428) and ANA-negative (titer <1:80, n = 250) groups. ANA-positive patients were more likely to be female (89.3% vs. 80.0%, p = 0.001) and to have early-onset RA (onset age <50 years: 67.6% vs. 59.6%, p = 0.035), suggesting a potential association between ANA positivity, female sex, and earlier disease onset (**Supplementary Table 1**). After PSM for age, sex, age at disease onset, and BMI, 247 matched pairs were analyzed. Post-matching comparisons revealed a significantly lower prevalence of alcohol consumption among ANA-positive patients (1.8% vs. 7.7%, p = 0.001) (**Supplementary Table 1**).

Comparative analysis of laboratory parameters, disease activity indices, and clinical manifestations between the two groups is presented in **Table 1**. ANA-positive patients had significantly lower lymphocyte counts, higher RF and anti-CCP positivity, and elevated IgG levels. Clinically, joint deformities were more frequent in ANA-positive individuals (29.5% vs. 19.0%, p = 0.017). Among extra-articular manifestations, hematologic involvement was significantly more common in the ANA-positive group (4.9% vs. 0.8%, p = 0.007), whereas other manifestations did not differ significantly between groups.

**Table 1:** Baseline laboratory parameters, disease activity, and clinical features between ANA-positive and ANA-negative RA patients.

Parameter	ANA-positive (n=247)	ANA-negative (n=247)	P-value
Laboratory parameters			
WBC (×10^9^/L)	6.47 (5.31,8.38)	6.92 (5.58,9.12)	0.058
Hemoglobin (g/L)	125 (117,134)	126 (116,136)	0.648
Platelets (×10^9^/L)	253 (217,310)	260.5 (213.5,319.3)	0.712
ALT (U/L)	18.00 (13.00,26.00)	18.00 (12.75,25.25)	0.932
AST (U/L)	19.00 (16.00,25.25)	21.00 (17.60,24.85)	0.344
Creatinine (μmol/L)	56.00 (48.00,65.50)	57.00 (50.10,66.00)	0.543
RF positivity, n (%)	194 (83.3%)	158 (65%)	<0.001
Anti-CCP positivity, n (%)	202 (86.7%)	154 (63.4%)	<0.001
GPI positivity, n (%)	16 (9.5%)	21 (12.0%)	0.460
ESR (mm/h)	27.00 (14.50, 49.50)	21.00 (11.5,53.50)	0.093
CRP (mg/L)	5.2 (2.00,13.89)	5.13 (0.67,21.90)	0.896
IgG (g/L)	15.35 (12.93,18.15)	13.85 (11.70,16.20)	<0.001
IgA (g/L)	2.88 (2.20, 3.65)	2.79 (2.13, 3.44)	0.145
IgM (g/L)	1.40 (1.01,1.92)	1.32 (0.95,1.84)	0.374
C3 (g/L)	1.13 (1.01,1.31)	1.17 (1.03,1.35)	0.199
C4 (g/L)	0.26 (0.21,0.31)	0.26 (0.21,0.33)	0.143
Disease activity and clinical outcomes			
TJC28	5 (2,10)	5 (2,10)	0.736
SJC28	2 (0,4)	2 (0,4)	0.373
Activity limitation, n (%)	147 (83.5%)	156 (78.0%)	0.177
Morning stiffness, n (%)	45 (25.6%)	41 (20.5%)	0.243
Joint deformities, n (%)	52 (29.5%)	38 (19.0%)	0.017
Functional disability, n (%)	8 (4.5%)	2 (1.0%)	0.070
PtGA (VAS, mm)	50 (30,70)	50 (30,60)	0.952
PhGA (VAS, mm)	40 (30,50)	40 (30,50)	0.456
DAS28-ESR	4.54 (3.71,5.59)	4.5 (3.52,5.58)	0.426
DAS28-CRP	4.27 (3.44,5.13)	4.25 (3.22,5.25)	0.941
SDAI	24.74 (15.50,44.30)	26.86 (15.54,50.13)	0.328
CDAI	16 (11,25)	16 (11,26)	0.840
HAQ-DI	0.625 (0.250,1.125)	0.625 (0.250,1.000)	0.665
EQ-5D	0.684 (0.505,0.826)	0.684 (0.542,0.783)	0.813
Extra-articular manifestations			
Interstitial lung disease (ILD), n (%)	18 (7.3%)	13 (5.3%)	0.354
Skin rash, n (%)	5 (2.0%)	8 (3.2%)	0.399
Ocular involvement, n (%)	4 (1.6%)	7 (2.8%)	0.360
Xerostomia (dry mouth), n (%)	4 (0.6%)	2 (0.8%)	0.681
Hematologic involvement, n (%)	12 (4.9%)	2 (0.8%)	0.007
Renal involvement, n (%)	14 (6.9%)	15 (6.8%)	0.944
Systemic symptoms*, n (%)	8 (3.2%)	16 (6.5%)	0.094

Data are presented as median (IQR) for continuous variables or n (%) for categorical variables. *Systemic symptoms include low-grade fever, dizziness, and fatigue not attributable to infection. Ocular involvement includes dry eye, conjunctivitis, and uveitis. Hematologic involvement includes leukopenia, anemia, and thrombocytopenia. Renal and hepatic involvement were determined by abnormal serum creatinine or elevated liver enzymes, respectively.

A total of 600 patients completed the 12-month follow-up, including 383 patients who were ANA-positive and 217 who were ANA-negative at baseline. After propensity score matching for sex, age, age at onset, baseline DAS28-ESR, DMARD regimen, and corticosteroid use, 214 matched pairs were retained for outcome analysis. As shown in **Table 2**, there were no significant differences between the ANA-positive and ANA-negative groups in terms of treatment or remission rates according to DAS28, SDAI, CDAI, Boolean, or ACR criteria, indicating comparable therapeutic responses.

**Table 2:** Comparison of treatment regimens and clinical outcomes between ANA-positive and ANA-negative RA patients.

Variable	ANA-positive (n = 214)	ANA-negative (n = 214)	P-value
Treatment Strategy			0.461
DMARDs use, n (%)			
csDMARDs	106 (49.5%)	106 (49.5%)	
bDMARDs	68 (31.8%)	59 (27.6%)	
tsDMARDs	40 (18.7%)	49 (22.9%)	
Glucocorticoid use, n (%)	113 (52.8%)	118 (55.1%)	0.628
Use of ≥2 csDMARDs, n (%)	72 (33.6%)	87 (40.7%)	0.133
Clinical outcomes at 12 months			
DAS28-ESR remission, n (%)	82 (38.3%)	89 (41.6%)	0.490
DAS28-CRP remission, n (%)	108 (50.0%)	106 (49.0%)	0.847
SDAI remission, n (%)	51 (23.8%)	46 (21.5%)	0.564
CDAI remission, n (%)	80 (37.4%)	72 (33.6%)	0.419
Boolean 1.0, n (%)	34 (15.9%)	32 (15.0%)	0.789
Boolean 2.0, n (%)	39 (18.2%)	41 (19.2%)	0.804
ACR20, n (%)	131 (61.2%)	129 (59.3%)	0.693
ACR50, n (%)	111 (51.9%)	107 (50.0%)	0.699
ACR70, n (%)	85 (39.7%)	69 (32.2%)	0.107
HAQ-DI	0.250 (0.125–0.500)	0.125 (0.000–0.375)	0.941
EQ-5D	0.869 (0.764–1.000)	0.869 (0.770–1.000)	0.935

Values are presented as median (IQR) for continuous variables or n (%) for categorical variables. p-values were derived from appropriate statistical tests (e.g., chi-square test, Fisher’s exact test, or Mann–Whitney U test). DMARDs, disease-modifying anti-rheumatic drugs; csDMARDs, conventional synthetic DMARDs; bDMARDs, biologic DMARDs; tsDMARDs, targeted synthetic DMARDs; DAS28, Disease Activity Score in 28 joints; CRP, C-reactive protein; SDAI, Simplified Disease Activity Index; CDAI, Clinical Disease Activity Index; HAQ-DI, Health Assessment Questionnaire Disability Index.

### Association between ANA development and clinical outcomes

**Figure 1:**
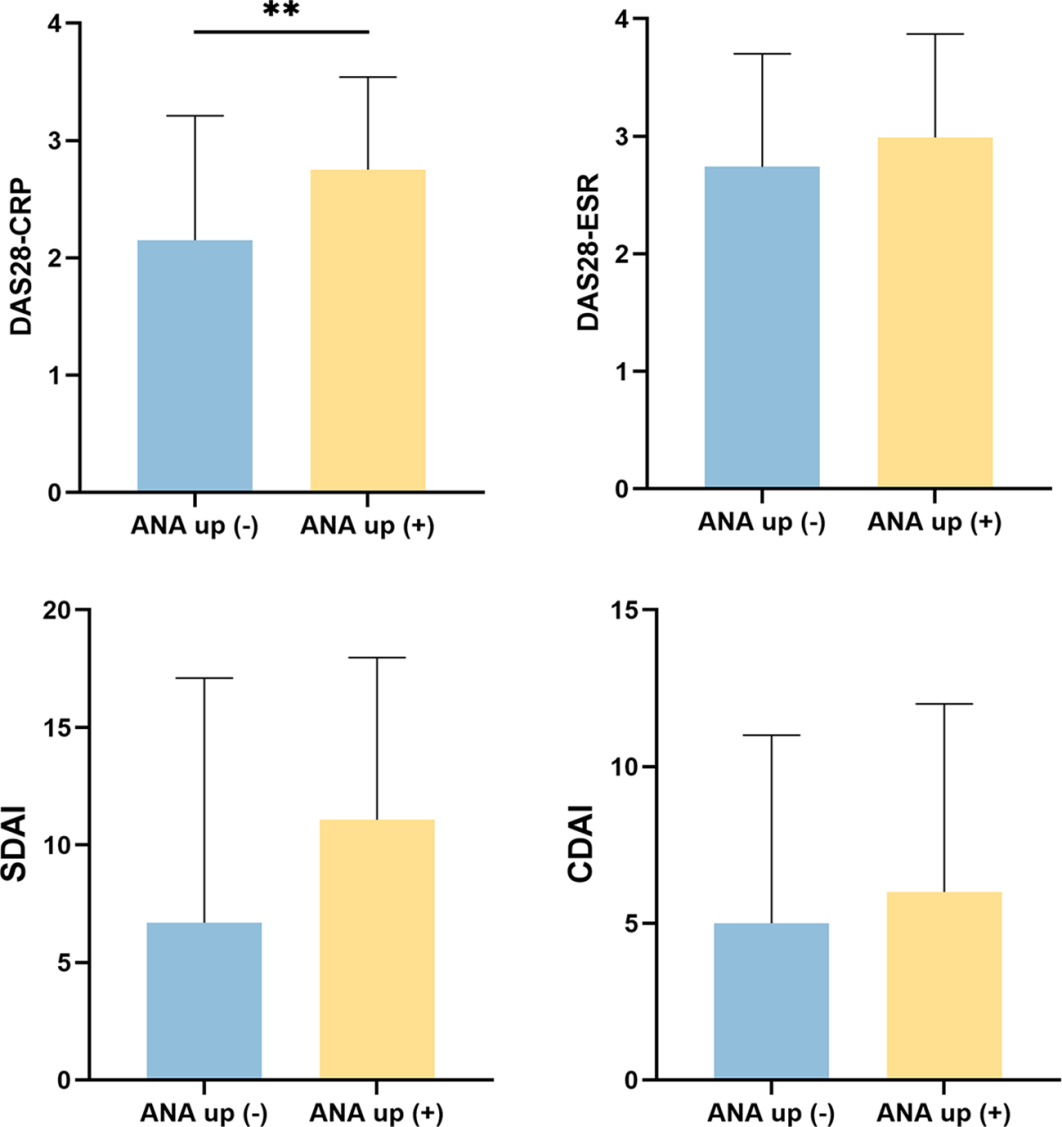
The histogram of follow-up disease activity score between the ANA-development and without ANA development group. This figure compares post-treatment disease activity scores—including DAS28-CRP, DAS28-ESR, SDAI, and CDAI—between patients with and without ANA titer elevation during follow-up. Patients with ANA development exhibited significantly higher disease activity scores, indicating poorer disease control. **P<0.01.

**Figure 2:**
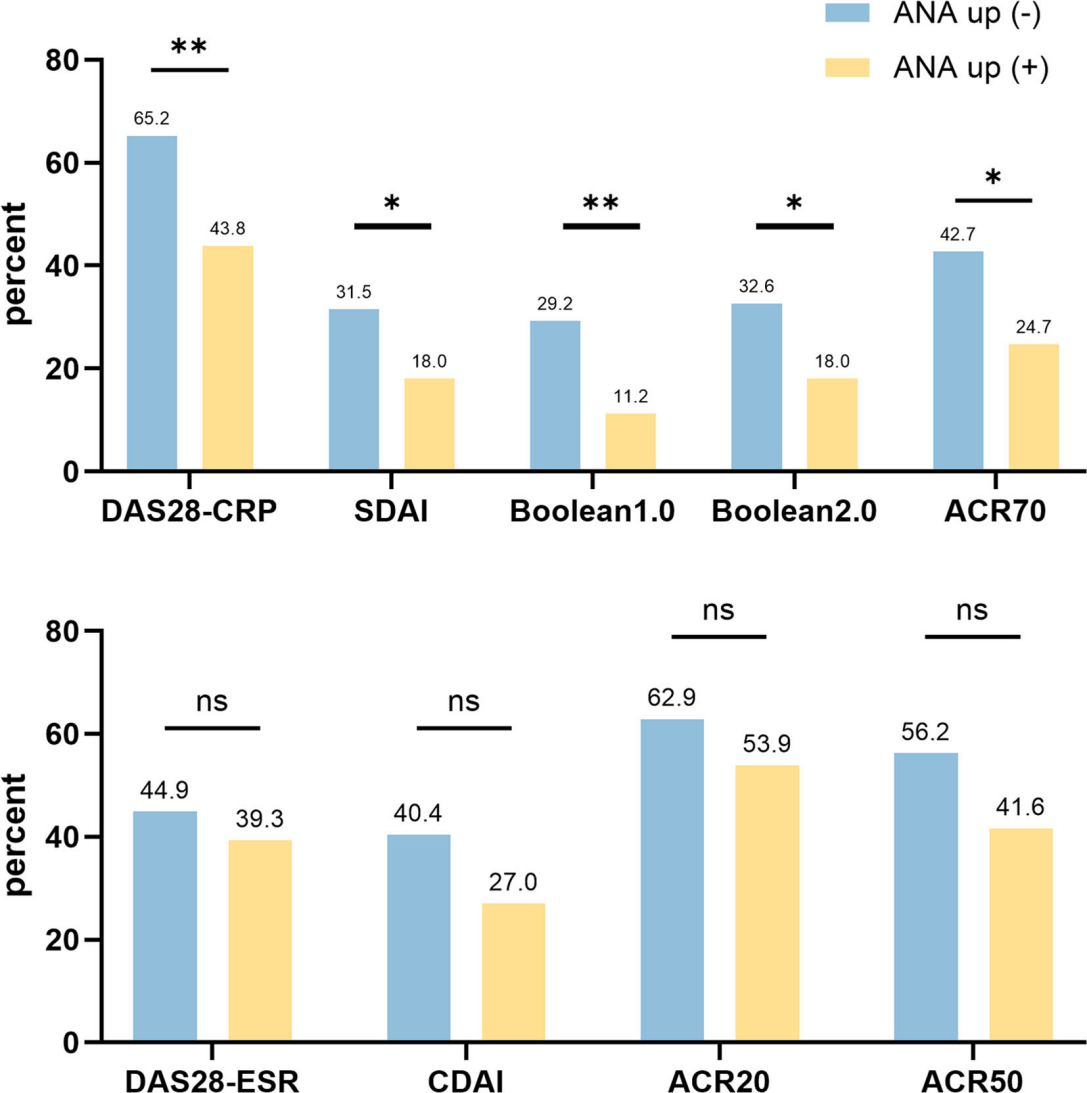
The histogram of remission rate between the ANA-development and without ANA development group. This figure illustrates the differences in remission rates across various clinical criteria (e.g., DAS28-CRP, SDAI, Boolean, ACR response) between patients with and without post-treatment ANA titer elevation. Statistically significant reductions in remission rates were observed in the ANA-development group. **p<0.01; *p<0.05.

At follow-up, ANA titers were re-evaluated in 467 patients. Of these, 94 patients (20.1%) exhibited an increase in ANA titer, while 373 patients (79.9%) had stable or decreased titers (**Supplementary Figure 3**). As shown in **Figure 1**, patients with ANA development had significantly higher post-treatment DAS28-CRP scores (median: 2.75 [2.01–3.54] vs. 2.15 [1.39–3.21], p = 0.007). Moreover, ANA titer elevation was associated with lower remission rates across multiple clinical criteria, including DAS28-CRP (43.8% vs. 65.2%, p = 0.004), SDAI (18.0% vs. 31.5%, p = 0.003), Boolean 1.0 (11.2% vs. 29.2%, p = 0.003), Boolean 2.0 (18.0% vs. 32.6%, p = 0.025), and ACR70 (24.7% vs. 42.7%, p = 0.011) (**Figure 2**). These findings suggest that an increase in ANA titer during treatment may be a negative prognostic marker for achieving clinical remission in RA. Furthermore, we compared the baseline disease activity levels between the ANA development and stable groups to assess potential confounding. No significant difference was found in the distribution of baseline disease activity (χ² = 3.192, p = 0.363), with 68.1% and 75.6% of patients exhibiting moderate-to-high disease activity, respectively.

### Association between treatment regimens and ANA levels

Previous studies have indicated that TNFi may trigger the development of ANA. To assess whether ANA development observed in our cohort was associated with specific therapeutic regimens, we stratified the 467 patients with complete follow-up data into three groups based on their treatment strategy: conventional synthetic DMARDs (csDMARDs), bDMARDs, and targeted synthetic DMARDs (tsDMARDs). To specifically isolate the effect of TNFi, patients receiving non-TNFi bDMARDs were excluded, resulting in 102 patients categorized into the bDMARDs (TNF inhibitors) group. To minimize confounding, PSM was performed based on disease duration, age at disease onset, and baseline DAS28-CRP. After matching, a total of 309 patients were included in the final analysis: 102 treated with csDMARDs, 102 with bDMARDs (TNF inhibitors), and 105 with tsDMARDs.

Post-treatment ANA profiles across the three groups are summarized in **Table 3**. Although the overall ANA positivity rates after treatment did not significantly differ among the three groups (p > 0.05), patients in the bDMARDs (TNF inhibitors) group demonstrated a significantly higher proportion of high ANA titers compared to those in the csDMARD group. Specifically, the proportion of patients with ANA titers ≥1:160 was significantly higher in the bDMARDs (TNF inhibitors) group (36.3% vs. 18.6%, p = 0.018), as were titers ≥1:320 (19.6% vs. 7.8%, p = 0.049) and ≥1:640 (10.8% vs. 2.0%, p = 0.031). However, the incidence of post-treatment ANA titer elevation was comparable across the csDMARD, bDMARDs (TNF inhibitors), and tsDMARD groups (p > 0.05). These findings suggest that although TNFi treatment may be associated with higher post-treatment ANA titers, the likelihood of ANA development is similar across therapeutic classes.

**Table 3:** Post-treatment ANA status across different DMARD regimens.

Variable	csDMARD group (n = 102)	bDMARDs (TNF inhibitors)^†^ (n = 102)	tsDMARD group (n = 105)	P-value
Post-treatment ANA positivity (≥1:80), n (%)	75 (73.5%)	77 (75.5%)	80 (76.2%)	0.901
Post-treatment ANA titer, n (%)				
1:80≤ANA<1:320	19 (18.6%)^a^	37 (36.3%)	28 (26.7%)	0.018
1:320≤ANA<1:640	8 (7.8%)^a^	20 (19.6%)	14 (13.3%)	0.049
ANA≥1:640	2 (2.0%)^a^	11 (10.8%)	6 (5.7%)	0.031
ANA development^*^	18 (17.6%)	20 (19.6%)	27 (25.7%)	0.331

Values are presented as n (%). P-values were derived using chi-square or Fisher’s exact test as appropriate.

a, Significantly different from the bDMARDs group (p < 0.05).

*, ANA development defined as elevation in ANA titer compared to baseline.

†, bDMARDi indicates TNF inhibitor–based biologic DMARDs.

### Prognostic model for DAS28-CRP remission

To predict treatment outcomes, we developed a multivariable logistic regression model with DAS28-CRP remission as the dependent variable. All 467 patients were classified into csDMARD, bDMARD, or tsDMARD treatment groups and randomly split into a training cohort (80%) and a testing cohort (20%) (**Supplementary Table 2**). Candidate predictors were identified using univariate logistic regression (**Supplementary Table 3**), and significant variables were then included in a multivariable stepwise regression analysis.

As shown in **Table 4**, ANA titer development following treatment (OR = 0.472, p = 0.010) and baseline DAS28-CRP (OR = 0.745, p = 0.017) were independent predictors of non-remission. Other contributing factors included patient age, alcohol consumption, and treatment regimen.

**Table 4:** Multivariate logistic regression analysis of predictors of remission in rheumatoid arthritis.

Variable	β coefficient	Wald χ²	P-value	OR (95% CI)
ANA development^*^	-0.750	6.559	0.010	0.472 (0.266, 0.839)
dsDNA positivity	0.295	0.246	0.620	1.344 (0.418, 4.320)
Age	-0.026	2.108	0.146	0.974 (0.941, 1.009)
Age at disease onset	0.015	0.708	0.400	1.015 (0.981, 1.049)
Higher education (≥Bachelor)	0.015	0.003	0.956	1.015 (0.592, 1.743)
Alcohol consumption	0.712	3.536	0.060	2.038 (0.970, 4.282)
Treatment (ref = csDMARDs)		7.662	0.022	
bDMARDs	-0.833	7.647	0.006	0.435 (0.241, 0.785)
tsDMARDs	-0.443	2.182	0.140	0.642 (0.357, 1.156)
Low-dose glucocorticoid use	-0.074	0.078	0.780	0.928 (0.550, 1.565)
Use of ≥2 csDMARDs	0.715	0.862	0.353	2.044 (0.452, 9.244)
Pain score	-0.011	2.397	0.172	0.989 (0.976, 1.003)
Baseline DAS28-CRP	-0.295	5.691	0.017	0.745 (0.585, 0.949)
HAQ-DI	0.083	0.132	0.716	1.087 (0.694, 1.702)
ESR	-0.001	0.057	0.812	0.999 (0.991, 1.007)
Hemoglobin	0.008	0.836	0.361	1.008 (0.991, 1.025)
Constant	2.056	2.597	0.107	7.818

*ANA development defined as elevation in ANA titer compared to baseline. OR, odds ratio; CI, confidence interval; ANA, antinuclear antibody; bDMARD, biologic DMARD; tsDMARD, targeted synthetic DMARD; csDMARD, conventional synthetic DMARD; DAS28-CRP, Disease Activity Score in 28 joints using C-reactive protein; HAQ-DI, Health Assessment Questionnaire Disability Index.

A final predictive model was constructed incorporating baseline DAS28-CRP, post-treatment ANA development, age, alcohol use, and treatment category. The logistic regression equation was as follows:


$$\begin{array}{l} logit\left(R\right)\;=\;2.788\;-\;0.622\;\times \;\left(ANA-development\right)\;-\;0.396\\ \times \;\left(baseline\;DAS28-CRP\right)\;-\;0.015\;\times \;\left(age\right)\;+\;0.794\\ \times \;\left(alcohol\;use\right)\;-\;0.543\;\times \;\left(bDMARDs\right)\;-\;0.252\\ \times \;\left(tsDMARDs\right) \end{array}$$

This model was visualized using a nomogram (**Supplementary Figure 4**). In the training cohort, the optimal cutoff value for predicted remission was 0.628, yielding a sensitivity of 68.2% and a specificity of 65.1%. The area under the ROC curve (AUC) was 0.715 (95% CI: 0.664–0.766, p < 0.001, **Supplementary Figure 5a**), indicating good discriminative ability. The Hosmer–Lemeshow goodness-of-fit test yielded a χ² of 5.403 (p = 0.714, **Supplementary Figure 5b**), and the calibration curve (**Supplementary Figure 6a**) demonstrated excellent agreement between predicted and observed outcomes.

In the testing cohort (n = 94), the AUC was 0.705 (95% CI: 0.658–0.751, p < 0.001), with similarly favorable calibration (Hosmer–Lemeshow χ² = 8.607, p = 0.377, **Supplementary Figure 6b**). Model performance metrics included an accuracy of 66.4%, sensitivity of 66.9%, specificity of 65.7%, positive predictive value (PPV) of 70.0%, negative predictive value (NPV) of 62.5%, a positive likelihood ratio of 1.953, and a negative likelihood ratio of 0.503. Collectively, these findings suggest that the developed model demonstrates acceptable calibration and moderate discrimination for predicting DAS28-CRP remission in RA patients.

### Subgroup analysis: distinguishing ANA seroconversion from titer elevation and sensitivity to higher threshold

To clarify the clinical significance of different dynamic changes, we subdivided patients with ANA development (n = 94) into two mutually exclusive subgroups: (1) ANA seroconversion (baseline negative to follow-up positive, n = 80) and (2) ANA titer elevation (baseline positive with a ≥1-dilution increase, n = 14). As shown in **Table 5**, both subgroups exhibited significantly lower DAS28-CRP remission rates (50% and 14.3%, respectively) compared to the ANA stable/decreased group (56.8%, overall p < 0.005). There was no significant difference in remission rates between the seroconversion and titer elevation subgroups (p > 0.05), suggesting that any form of ANA emergence or increase portends a poorer therapeutic outcome.

**Table 5:** Subgroup analysis and sensitivity analyses.

Variable	n/N (%)	DAS28-CRP remission, n (%)	Statistical method	Effect estimate (95% CI) or p-value
ANA development patterns	467/467 (100)		Pearson χ²	0.005
ANA stable/decreased	373 (79.9)	212 (56.8)	Reference	–
ANA seroconversion^*^	80 (17.1)	40 (50.0)	Pearson χ² vs. stable	0.264
ANA titer elevation^†^	14 (3.0)	2 (14.3)	Pearson χ² vs. stable	0.002
ANA Positivity Threshold	467/467 (100)		Logistic regression	OR=1.62 (1.10-2.41); p = 0.016
ANA titer <1:160	313 (67.0)	158 (50.5)	Reference	1.00
ANA titer ≥1:160	154 (33.0)	96 (62.3)	Unadjusted model	1.62 (1.10-2.41)

*ANA seroconversion defined as change from negative (<1:80) to positive (≥1:80) at follow-up.

†ANA titer elevation defined as ≥1 two-fold dilution increase from baseline positive titer.

Sensitivity analysis using a higher ANA positivity threshold (≥1:160) revealed that baseline high-titer ANA was associated with better treatment response(OR = 1.624, 95% CI: 1.095-2.408, p = 0.016), emphasizing that dynamic changes during treatment rather than static baseline titers carry prognostic significance. Furthermore, we compared the baseline disease activity levels between the ANA development and stable groups. There was no significant difference in the distribution of baseline disease activity between the two groups (p = 0.363), with 68.1% and 75.6% of patients exhibiting moderate-to-high disease activity, respectively.

### Stratified analysis by baseline RF and anti-CCP status

Given the association between baseline ANA positivity and higher RF/anti-CCP levels, we investigated whether the prognostic value of ANA development was modulated by these classic RA autoantibodies. Stratified multivariate analyses (adjusted for the same 15 covariates as the main model) revealed a differential modulation effect (**Table 6**). In RF-negative patients, ANA development showed the strongest association with non-remission (adjusted OR = 0.29, 95% CI: 0.09-0.99, p = 0.048). In contrast, this association was attenuated and non-significant in RF-positive patients (OR = 0.59, 95% CI: 0.31-1.09, p = 0.092). For anti-CCP, a significant association was maintained in anti-CCP-positive patients (OR = 0.53, 95% CI: 0.28-0.98, p = 0.043), with a strong trend in anti-CCP-negative patients (OR = 0.29, p = 0.054). This suggests that the prognostic weight of ANA dynamics is context-dependent, being particularly salient in RF-negative patients. Baseline serological characteristics are summarized in **Table 3**. While RF and anti-CCP status individually showed no significant association with remission rates (RF-positive vs. negative: 54.7% vs. 52.8%, p = 0.729; anti-CCP-positive vs. negative: 52.8% vs. 59.6%, p = 0.227), combined analysis revealed heterogeneity. Notably, patients who were RF-positive but anti-CCP-negative had the highest remission rate (73.0%, 27/37), significantly exceeding the double negative group (51.6%, 32/62; OR = 2.53, p = 0.034).

**Table 6:** Association of baseline serological status with DAS28-CRP remission.

Variable	n/N (%)	DAS28-CRP remission, n (%)	Unadjusted OR (95% CI)	p-value^†^
RF Status^*^	461/467 (98.7)			
RF-negative	108 (23.4)	57 (52.8)	Reference	–
RF-positive	353 (76.6)	193 (54.7)	1.08 (0.70-1.66)	0.729
Anti-CCP Status^*^	461/467 (98.7)			
Anti-CCP-negative	99 (21.5)	59 (59.6)	Reference	–
Anti-CCP-positive	362 (78.5)	191 (52.8)	0.76 (0.48-1.19)	0.227
Combined Serology	461/467 (98.7)		χ²=5.775, p=0.123	Combined Serology
Double negative (RF-/CCP-)	62 (13.4)	32 (51.6)	Reference	–
RF-negative/CCP-positive	46 (10.0)	25 (54.3)	1.12 (0.52-2.41)	0.775
RF-positive/CCP-negative	37 (8.0)	27 (73.0)	2.53 (1.07-5.99)	0.034
Double positive (RF+/CCP+)	316 (68.5)	166 (52.5)	1.04 (0.60-1.81)	0.889

*Six patients had missing RF or anti-CCP data (461/467 with complete serology).

†p-values for pairwise comparisons against the double negative reference group (Fisher’s exact test).

All stratified models adjusted for 15 covariates: age, age at onset, education level, alcohol use, baseline DAS28-CRP, baseline HAQ, ESR, hemoglobin, dsDNA positivity, and treatment variables (bDMARDs, tsDMARDs, csDMARDs ≥2, glucocorticoids).

To clarify the independent prognostic value of ANA dynamics, we constructed a parsimonious model adjusting for RF and anti-CCP status (**Supplementary Table 4**). ANA development remained significantly associated with lower remission rates (adjusted OR = 0.57, 95% CI: 0.36-0.90, p = 0.017), independent of serological status. However, this association was attenuated when ANA patterns were included in the full model (**Table 4**), suggesting that the underlying fluorescence pattern may mediate part of the effect of ANA titer changes.

### Analysis of ANA immunofluorescence patterns and treatment response

We analyzed four common ANA fluorescence patterns: homogeneous, speckled, centromere, and plasmicspeck. To avoid confounding from co-existing patterns, patients were classified into mutually exclusive groups (**Table 7**). The distribution was: pure homogeneous (n = 116, 24.8%), pure speckled (n = 94, 20.1%), pure plasmicspeck (n = 20), pure centromere (n = 18), mixed patterns (n = 36, 7.7%), and all negative (n = 183, 39.2%). Remission rates differed significantly across groups (χ² = 16.480, p = 0.006). Strikingly, patients with a pure homogeneous pattern had the highest remission rate among the major pattern groups (63.8%, 74/116), significantly higher than those with a pure speckled pattern (41.5%, 39/94; p<0.001). The mixed pattern group showed the highest remission rate (72.2%), though sample size was limited. In multivariate logistic regression including all four patterns, the homogeneous pattern remained independently associated with remission (adjusted OR = 1.94, 95% CI: 1.28-2.95, p = 0.002), while other patterns showed no significant independent effects (**Supplementary Table 5**).

**Table 7:** DAS28-CRP remission by mutually exclusive ANA pattern groups.

Pattern group	Total N (%, of 467)	Remission (n, %)	Non-remission (n, %)
Pure Homogeneous	116 (24.8%)	74 (63.8%)	42 (36.2%)
Pure Speckled	94 (20.1%)	39 (41.5%)	55 (58.5%)
Pure PlasmicSpeck	20 (4.3%)	12 (60.0%)	8 (40.0%)
Pure Centromere	18 (3.9%)	8 (44.4%)	10 (55.6%)
Mixed Patterns (≥2)	36 (7.7%)	26 (72.2%)	10 (27.8%)
All Negative	183 (39.2%)	95 (51.9%)	88 (48.1%)
Total	467 (100%)	254 (54.4%)	213 (45.6%)

χ²=16.480, p=0.006.

## Discussion

Our findings prompt a critical re-evaluation of the clinical utility of serial antinuclear antibody (ANA) monitoring in rheumatoid arthritis (RA) management. Current guidelines do not endorse routine ANA follow-up in RA, as ANA is conventionally regarded as a static diagnostic marker rather than a dynamic biomarker of disease activity (22). However, the strong association observed in our cohort between rising ANA titers and poor treatment response suggests that increasing titers may serve as an early warning indicator of therapeutic failure. Historically, research on ANA kinetics has predominantly focused on systemic lupus erythematosus (SLE). In RA, the immunopathogenic relevance of ANA is less established, given that disease development is centered on citrullinated antigens and epitope spreading rather than ANA-driven systemic autoimmunity. Nevertheless, RA-specific evidence is accumulating. Ishikawa et al. reported that ANA development in RA patients receiving biologic disease-modifying anti-rheumatic drugs (bDMARDs) was significantly associated with poor treatment response, suggesting that dynamic changes in ANA may reflect immune activation linked to treatment resistance (19, 20, 23). Furthermore, broader studies on autoantibody dynamics—such as the finding by Brevet et al. (24) that restriction of ACPA fine specificities was associated with clinical response to abatacept and methotrexate—further support the value of monitoring the evolution of the autoantibody repertoire in RA. Although ACPA and ANA represent distinct autoantibody systems, these findings collectively suggest that focusing on the dynamic evolution of autoantibody profiles, rather than relying solely on static measurements, may carry clinical utility. Our results align with this concept, demonstrating that rising ANA titers during treatment are associated with significantly lower remission rates. Nevertheless, larger longitudinal RA studies with extended follow-up are required to confirm the prognostic significance of ANA dynamics and to establish how best to incorporate this parameter into clinical practice (25).

Consistent with prior reports (16, 17, 26), we confirmed that ANA-positive RA patients are predominantly female and exhibit higher levels of RF, anti-CCP antibodies, and γ-globulin. More importantly, our stratified analysis demonstrated that the prognostic impact of rising ANA titers is not uniform but is significantly influenced by the baseline autoantibody profile. The association was most pronounced in RF-negative patients, indicating that ANA dynamics might serve as a particularly useful prognostic tool in this seronegative subset, which is often more heterogeneous and lacks reliable predictive biomarkers. The differential modulation by RF and anti-CCP—where RF positivity attenuated the ANA effect more strongly than anti-CCP positivity—suggests involvement of distinct immunological pathways. A noteworthy secondary finding was the higher remission rate in the RF-positive/anti-CCP-negative subgroup. This serological profile, found in 8.0% of our cohort, may identify a distinct RA phenotype with differential treatment responsiveness. The dissociation between RF and anti-CCP in these patients merits further study to elucidate the underlying immunopathogenesis. In addition, we observed a significantly higher prevalence of joint deformities among ANA-positive patients, further supporting the notion that ANA positivity may be associated with a more severe or erosive disease phenotype. Although some studies have reported associations between ANA positivity and higher swollen joint counts or evaluator global assessment (EGA) scores (27), we found no significant differences in baseline composite disease activity indices—such as DAS28, SDAI, or CDAI—between ANA-positive and ANA-negative groups. This discrepancy implies that ANA status may not directly correlate with acute joint inflammation but may instead reflect cumulative disease burden or systemic immune activation.

A novel and unexpected finding was association between specific ANA fluorescence patterns and treatment outcomes. Contrary to the negative prognostic implication of rising ANA titers, the presence of a baseline homogeneous pattern was associated with a significantly higher likelihood of achieving remission compared to a speckled pattern. This pattern-dependent effect persisted in a multivariate model, suggesting an independent signal. The homogeneous pattern is often linked to antibodies against dsDNA or histones, while the speckled pattern is associated with anti-ENA antibodies (e.g., anti-SSA/Ro). The reasons for this divergent prognostic value are unclear but may relate to differences in the underlying B-cell or T-helper cell responses they represent. Interestingly, patients with mixed patterns had the highest remission rate, though this group was small. These findings suggest that static ANA patterns might identify RA subsets with different biological backgrounds and treatment responsiveness, adding complexity to the interpretation of ANA in RA.

Sensitivity analysis using a higher titer cutoff (≥1:160) introduced a seemingly paradoxical finding: while an increase in ANA titer predicted poorer outcomes, a high baseline titer (≥1:160) was associated with a higher likelihood of remission. This divergence highlights a critical distinction between static autoimmune status and dynamic immunologic shifts. A high, stable baseline ANA titer might represent a distinct, treatment-responsive RA endotype, frequently associated with the homogeneous pattern which we found to be prognostically favorable. Conversely, a longitudinal rise in ANA titer—regardless of the starting point—likely signals an active, evolving immune response or treatment-induced immunomodulation (e.g., secondary to TNF inhibitors), which may interfere with the achievement of clinical remission. Therefore, our findings suggest that the dynamic trajectory of ANA levels, rather than a single baseline snapshot, provides more actionable prognostic information in RA.

TNF inhibitors (TNFi) are well known to induce ANA and other autoantibodies in a subset of RA patients (28, 29). In our cohort, while TNFi treatment was associated with higher absolute ANA titers post-treatment, the incidence of ANA development was statistically similar across different therapeutic classes (p > 0.05). Importantly, our multivariable model adjusted for treatment regimens and confirmed that ANA development remained an independent predictor of non-remission. This suggests that the observed association is not merely a secondary effect of TNFi therapy but likely reflects a broader immunological shift linked to treatment resistance. However, since different biologics have varying immunogenic potentials, future studies should address whether dynamic ANA changes can act as early biomarkers for treatment failure, secondary autoimmunity, or progression to overlap syndromes in at-risk individuals.

The limitations of this study include its single-center design and the predominantly East Asian study population, which may limit the generalizability of the findings to other ethnic or geographic groups. Additionally, the retrospective nature meant that 6-month disease activity scores were not available for all patients; however, the comparable baseline disease activity distributions between the groups (p = 0.363) suggest that the observed association carries prognostic weight independent of initial severity. The lack of comprehensive immunophenotyping—such as interferon signature, B-cell subset, or cytokine analyses—hindered exploration of the mechanisms behind ANA titer elevation. Furthermore, while we performed stratified and pattern analyses, some subgroups were small, which may affect the precision of those estimates. The pattern analysis, while informative, did not include data on antigen-specific antibodies (e.g., anti-dsDNA, anti-ENA) to confirm the associations mechanistically. Finally, the 12-month follow-up period may have been insufficient to capture long-term outcomes, including radiographic progression, extra-articular manifestations, or late treatment effects.

In summary, our results suggest that monitoring ANA titer dynamics—rather than relying solely on static ANA status—could provide valuable insights into disease progression and therapeutic responsiveness in RA. The prognostic utility appears strongest in RF-negative patients and can be further refined by incorporating baseline ANA fluorescence patterns. The seemingly paradoxical relationships between high baseline ANA titers and ANA titer increase highlight the nuanced role of ANA in RA. Accurate interpretation depends heavily on context, including coexisting autoantibodies, specific fluorescence patterns, and the nature of the titer change (static level versus dynamic rise). A rising ANA titer during treatment may therefore signal the need for closer surveillance, earlier intervention, or adjustment of therapeutic strategy.

## Data Availability

The original contributions presented in the study are included in the article/**Supplementary Material**. Further inquiries can be directed to the corresponding authors.
