# Supplementary material for: Prognostic value of longitudinal antinuclear antibody dynamics in rheumatoid arthritis: a retrospective cohort study
Source: Front Immunol. 2026 Apr 21;17:1743637. doi: 10.3389/fimmu.2026.1743637 (PMC13140284; doi:10.3389/fimmu.2026.1743637)
Supplement: Supplementary file 2 [file Supplementaryfile2.docx]

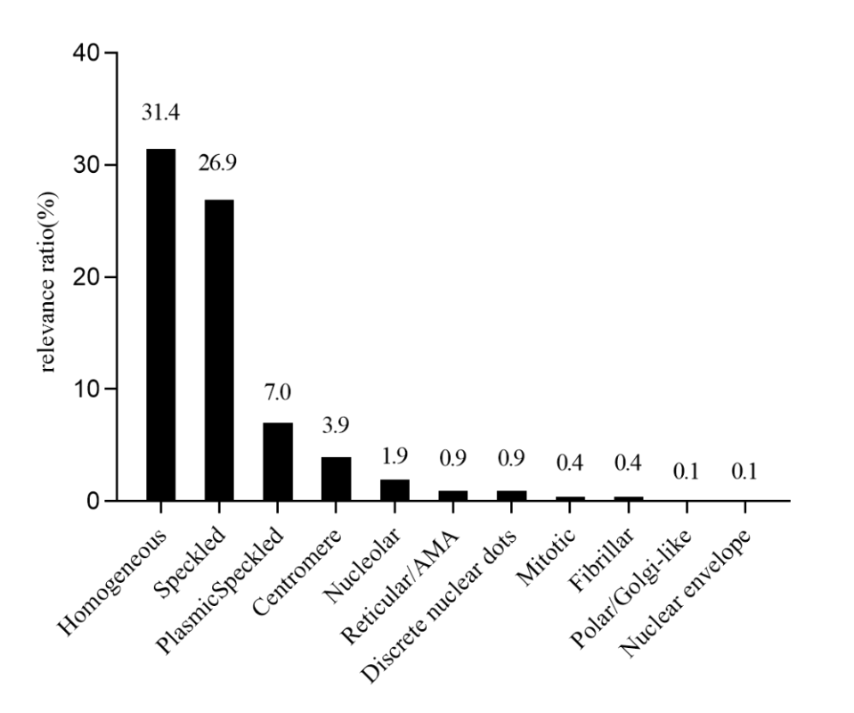


Fig.1 The positive rate of different ANA nuclear patterns in rheumatoid arthritis


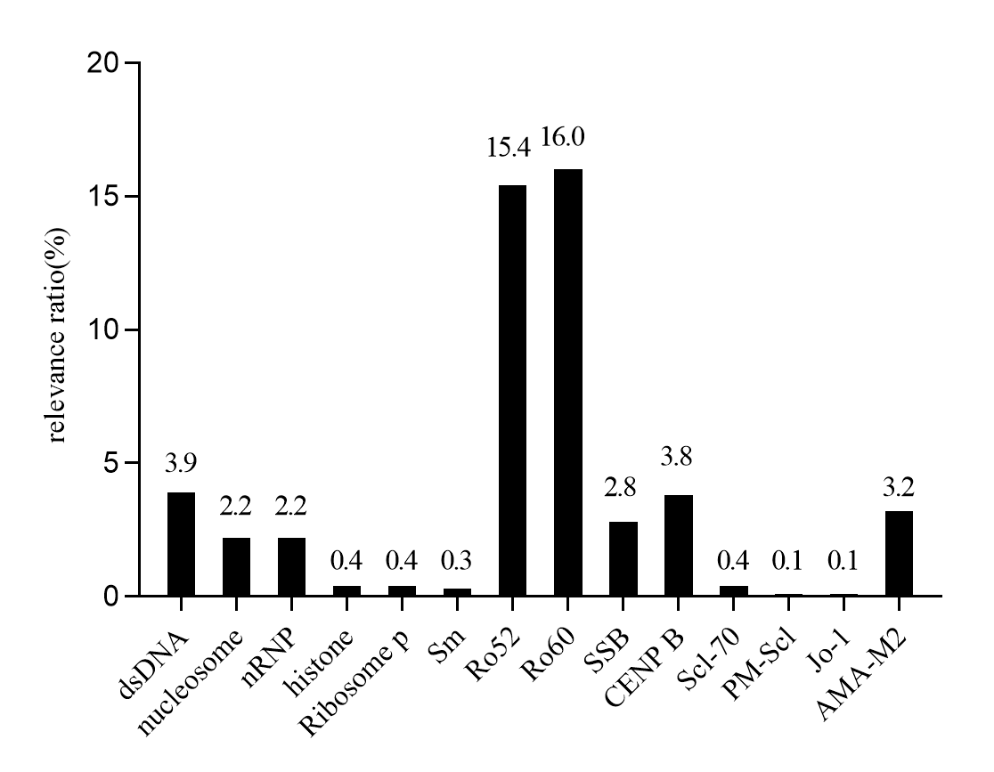


Fig.2 The positive rate of antinuclear antibody spectrum in rheumatoid arthritis


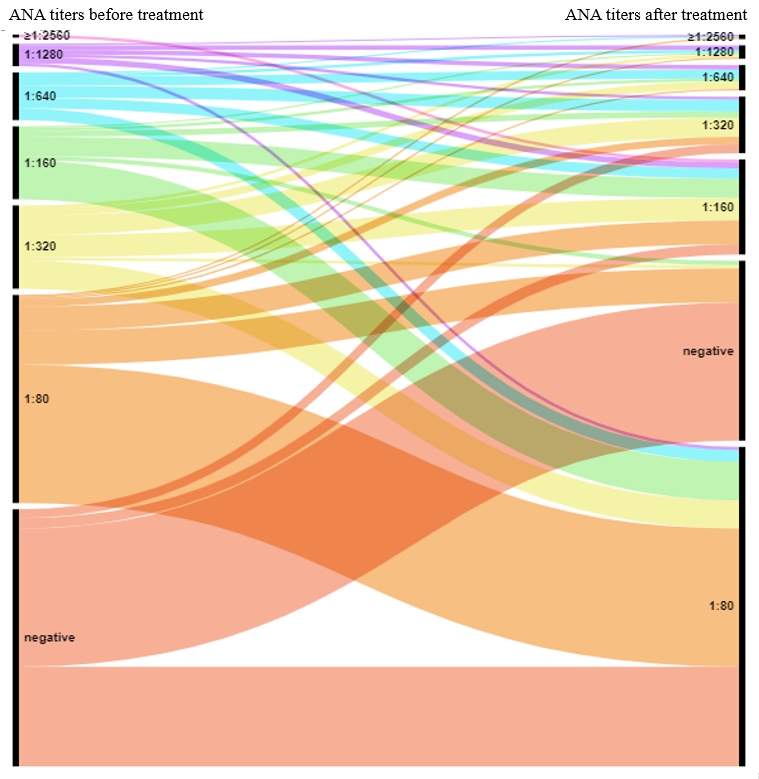


Fig.3 The Sankey diagram of ANA titers before and after treatment

Note:This diagram visualizes the dynamic changes in antinuclear antibody (ANA) titers from baseline to post-treatment among RA patients.


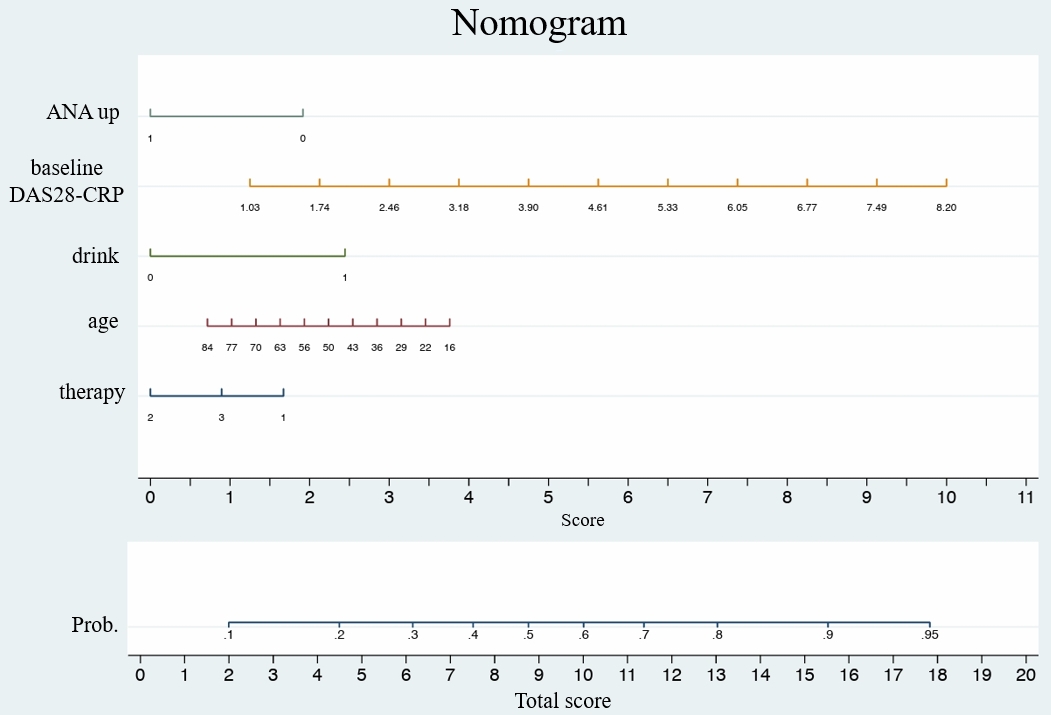


Fig.4 Nomogram of the multi-factor prediction model

Note: The nomogram integrates ANA titer elevation, baseline DAS28-CRP, age, alcohol consumption, and treatment strategy to estimate the probability of achieving remission. Each variable corresponds to a point on the top scale, and the total score maps to the predicted probability of remission on the bottom scale.


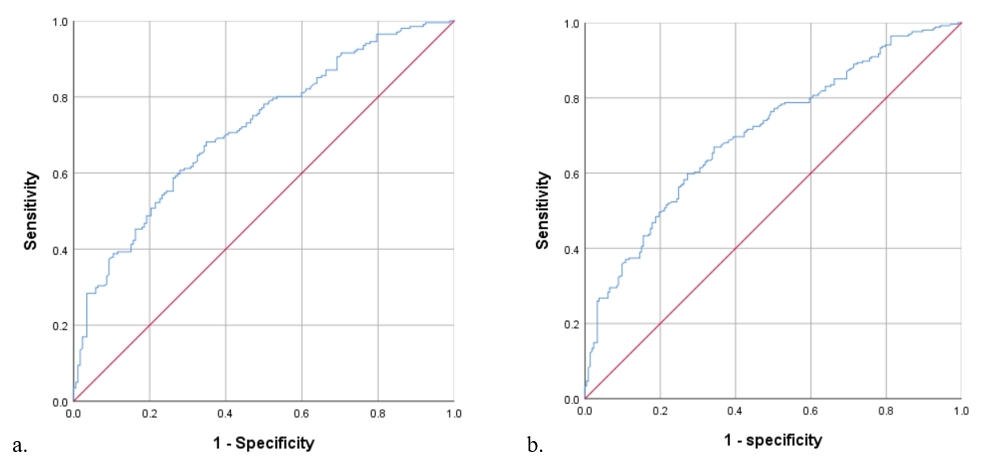


Fig.5 ROC curve of the multi-factor prediction model of training set and test set

Note:
(a) ROC curve for the training set.
(b) ROC curve for the test set.
The model demonstrated good discriminatory performance in both sets, with area under the curve (AUC) values indicating acceptable predictive ability for DAS28-CRP remission in RA.


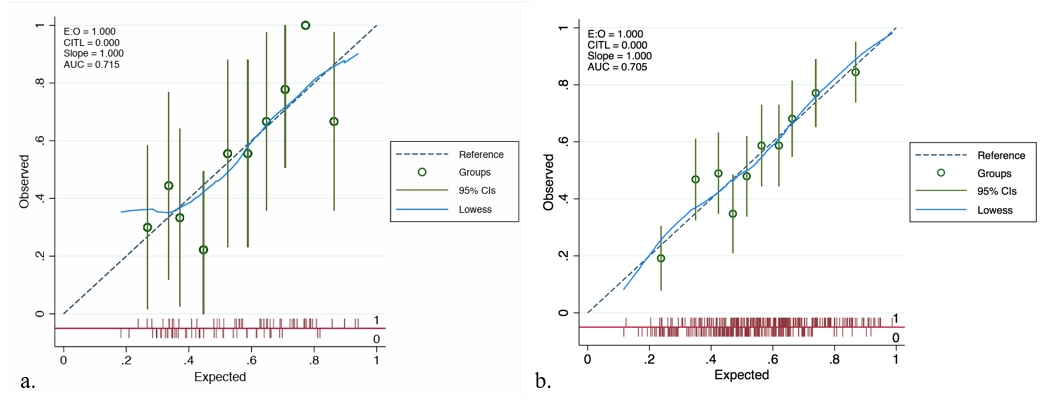


Fig.6 Calibration plot of the multi-factor prediction model of training set and test set

Note:
(a) Calibration plot for the training set.
(b) Calibration plot for the test set.
The plots compare predicted probabilities of DAS28-CRP remission with observed outcomes. The diagonal line represents perfect calibration. The model demonstrated good agreement between predicted and actual outcomes in both cohorts.
